# Efficacy and safety of early initiation of Sacubitril/Valsartan in patients after acute myocardial infarction: A meta‐analysis

**DOI:** 10.1002/clc.23717

**Published:** 2021-08-31

**Authors:** Jing Zhao, Yuanyuan Zeng, Xiaoxu Shen

**Affiliations:** ^1^ Cardiology Department Dongzhimen Hospital, Beijing University of Chinese Medicine Beijing China

**Keywords:** acute myocardial infarction, early initiation, meta‐analysis, Sacubitril/Valsartan

## Abstract

Some randomized controlled trials have compared the effectiveness and safety outcomes between early initiation of Sacubitril/Valsartan and angiotensin‐converting enzyme inhibitors (ACEIs) in patients after acute myocardial infarction. Therefore, our current meta‐analysis aimed to clarify the confusion. Four Databases and relevant grey literature were searched for studies from inception to July 2, 2021. Two reviewers independently screened literature, extracted data, and assessed the risk of bias. Four studies involving 6154 patients were included to perform meta‐analysis. The results of meta‐analysis showed that the left ventricular ejection fraction in the Sacubitril/Valsartan group was higher than the ACEI group (SMD: 0.37, 95% CI: 0.19–0.55, *P* = .000), the incidence of major adverse cardiac events in the Sacubitril/Valsartan group was lower than the ACEI group (RR: 0.61, 95% CI: 0.46–0.82, *P* = .001), while the incidences of cardiac death (RR: 1.00, 95% CI: 0.81–1.24, *P* = 1.000) and the heart failure hospitalization (RR: 0.62, 95% CI: 0.37–1.03, *P* = .065) showed no difference. For the incidences of myocardial infarction and the adverse side effects, there was no obvious advantage of the Sacubitril/Valsartan group over the ACEI group, because the meta‐analysis was not performed due to the limited trials. This study indicated that early initiation of Sacubitril/Valsartan in patients after acute myocardial infarction was superior to ACEI in reducing the risks of major adverse cardiac events and left ventricular ejection fraction increasing. As for the other outcomes (the incidences of cardiac death, the heart failure hospitalization, the myocardial infarction and the adverse side effects), Sacubitril/Valsartan showed no obvious advantage than ACEI.

## 1 INTRODUCTION

Despite the remarkable advances in the treatment of coronary artery disease and acute myocardial infarction (AMI) over the past two decades, AMI remains the most common cause of heart failure (HF).^1^ The development of HF increases total mortality risk three‐fold among patients with a history of MI.^2^ The activation of the renin–angiotensin–aldosterone system (RAAS) participates in the process of the left ventricle remodeling and HF development after AMI.^3^ Angiotensin‐converting enzyme inhibitors (ACEIs) and angiotensin receptor blockers (ARBs) inhibit the activation of RAAS by blocking the conversion of angiotensin I into angiotensin II and interfering with the binding of angiotensin II to its receptor, respectively. Early initiation of ACEI from MI symptom onset could reduce 30‐day mortality and HF by 7% and 4%, respectively.^4^ ARBs are used in patients with intolerance of ACEIs.^5^, ^6^

Sacubitril/Valsartan, which consists of the neprilysin inhibitor sacubitril and the ARB valsartan, has been approved for patients with symptomatic heart failure with reduced ejection fraction (HFrEF) and is intended to be substituted for ACEIs or ARBs.^7^, ^8^ In the PARADIGM‐HF study, Sacubitril/Valsartan in HFrEF patients reduced HF hospitalization by 20% compared with ACEI enalapril.^7^ The PIONEER‐HF study showed that early initiation of Sacubitril/Valsartan in MI patients with left ventricle systolic dysfunction could reduce the level of NT‐pro BNP.^9^ In recent years, many researchers focused on whether AMI patients benefit of early initiation of Sacubitril/Valsartan.^10^, ^11^, ^12^ However, there is still a lack of relevant clinical evidence. Therefore, we conducted this meta‐analysis to investigate the efficacy and safety of early initiation of Sacubitril/Valsartan in patients after AMI.

## 2 METHODS

Our current meta‐analysis was performed based on the Cochrane handbook for systematic reviews. The results of this study were arranged based on the Preferred Reporting Items for Reporting Systematic Reviews and Meta‐analyses (PRISMA). The data, methods, and materials of this study are available to others for purposes of reproducing the results or replicating procedures by contacting the corresponding author.

### 2.1 Search strategy

Databases including Web of Science, PubMed, Embase, and the Cochrane Library were searched for relevant studies from inception to July 2, 2021. The relevant grey literature, like reports and conference abstracts on the Internet, was also searched. The search terms were as follows: myocardial infarction, sacubitril‐valsartan, sacubitril valsartan sodium hydrate, LCZ 696, angiotensin receptor neprilysin inhibitor, and randomized controlled trial. All searches were performed independently by two reviewers. Discrepancies between reviewers were resolved by discussion or by a third reviewer.

### 2.2 Study eligibility

Eligible studies must meet the following criteria: (a) randomized controlled trial (RCT) focused on the patients after AMI occurred within 1 month as well as hemodynamics permit, (b) the comparisons of outcomes between Sacubitril/Valsartan and ACEIs, (c) the effectiveness outcomes included cardiac death, MI, HF hospitalization, major adverse cardiac events (MACE) and left ventricular ejection fraction (LVEF), the safety outcomes including adverse side effects.

The exclusion criteria were as follows: (a) studies were duplicated publications, (b) studies without useable data, (c) pediatric, animal or cell studies, (d) studies were published in non‐English or non‐Chinese.

### 2.3 Data extraction

Two investigators extracted the following data independently from each of the studies included: the first author, year of publication, study country, funding support, patient characteristics (age, sex ratio), interventions (grouping, sample size, types of drugs, intervention duration), outcomes. In the case of missing information in the included studies, investigators were contacted by email to obtain the missing information.

### 2.4 Quality assessment

Two independent reviewers assessed the risk of bias of all included trials and completed a Risk of Bias Table as described in chapter 8 of the Cochrane Handbook.^13^

### 2.5 Statistical analysis

The meta‐analysis was conducted by Stata 15.0 software. Heterogeneity between trial results was tested using the Q test and the *I* ^2^ statistic where percentages greater than 50% were taken to indicate significant heterogeneity. If heterogeneity was detected for outcomes, meta regression, subgroup analysis and sensitivity analysis were performed to analyze the causes of heterogeneity. The test level of meta regression was set as *α* = 0.1. Funnel plot was used to evaluate the publication bias. The test level of meta‐analysis was set as *α* = 0.05.

## 3 RESULTS

### 3.1 Search results and population characteristics

**FIGURE 1:**
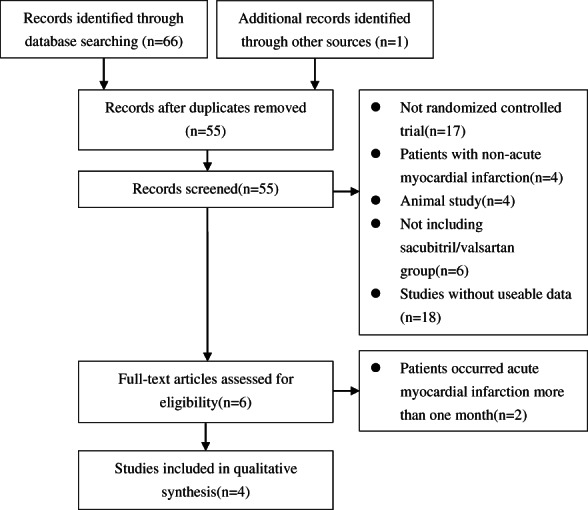
Study selection process

The study selection process was illustrated in Figure 1. We identified 66 records in the initial search. After removing duplicates and screening, four studies were eligible for inclusion in our meta‐analysis. The population characteristics of the patients were summarized in Table 1.

**TABLE 1:** Characteristics of the clinical trials included in the meta‐analysis

Study	Country	Patients	*n*	Age	Drugs	Duration	Funding
			(T/C)	(T/C, years old)	(T/C)		
Rezq^14^	Egypt	Patients with ST‐segment elevation myocardial infarction	100/100	52 ± 9.2/57 ± 11.6	Sacubitril‐Valsartan/ACEI	6 months	Yes
Wang^15^	China	Patients with left ventricular systolic dysfunction following acute anterior wall myocardial infarction	68/69	59.13 ± 7.15/60.56 ± 7.62	Sacubitril‐Valsartan/ACEI	6 months	Unclear
Zhang^16^	China	Patients with ST‐elevation myocardial infarction after primary percutaneous coronary intervention	79/77	60.3 ± 11.7/60.0 ± 10.9	Sacubitril‐Valsartan/ACEI	6 months	Unclear
Kunbhani^17^	41 countries	Acute myocardial infarction patients without known prior heart failure	2380/2381	64/64	Sacubitril‐Valsartan/ACEI	23 months	Yes

Abbreviations: ACEI, angiotensin‐converting enzyme inhibitor; C, ACEI group; T, Sacubitril/Valsartan group.

### 3.2 Quality assessment

The Cochrane Collaboration's tool for assessing risk of bias was used to assessing the bias of studies included (Table 2). Rezq^14^ declared that the random numbers were computer‐generated. Kunbhani^17^ declared that the patients were randomized via an interactive response technology. Only Wang^15^ noted that the patients were assigned by the envelope method.

**TABLE 2:** Summary of the quality assessment by The Cochrane Collaboration's tool of included studies

Study	Random sequence generation	Allocation concealment	Blinding of participants and personnel	Binding of outcome assessment	Incomplete outcome data	Selective reporting	Other bias
Rezq^14^	Low risk	Unclear risk	Low risk	Low risk	Low risk	Low risk	Unclear risk
Wang^15^	Unclear risk	Low risk	Unclear risk	High risk	Low risk	Low risk	Unclear risk
Zhang^16^	Unclear risk	Unclear risk	High risk	High risk	Low risk	Low risk	Unclear risk
Kunbhani^17^	Low risk	Unclear risk	Low risk	Unclear risk	Low risk	Low risk	Unclear risk

### 3.3 Cardiac death outcome in patients after AMI

**FIGURE 2:**
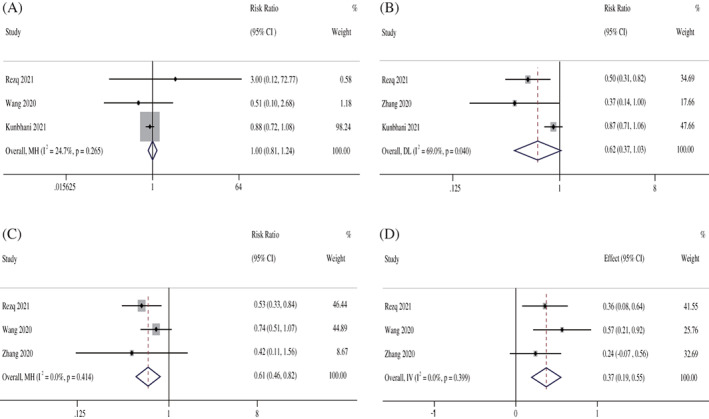
The forest plots of the effectiveness and safety outcomes between early initiation of Sacubitril/Valsartan and angiotensin‐converting enzyme inhibitors in patients after acute myocardial infarction. (A) The forest plots of the incidence of cardiac death, (B) the forest plots of the incidence of heart failure hospitalization, (C) the forest plots of the incidence of major adverse cardiac events, (D) the forest plots of the left ventricular ejection fraction

Three trials^14^, ^15^, ^17^ involving 5998 patients reported cardiac death outcome. The significant heterogeneity was not noted between the included studies (*I* ^2^ = 24.7%, *P* = .265). Therefore, the fixed‐effects M‐H model was used. The meta‐analysis showed that no significant difference in the incidence of cardiac death was noted between the Sacubitril/Valsartan group and the ACEI group (RR: 1.00, 95% CI: 0.81–1.24, *P* = 1.000; Figure 2(A)).

### 3.4 MI outcome in patients after AMI

Two trials^14^, ^15^ involving 337 patients reported myocardial infarction outcome. However, a meta‐analysis could not be performed due to the limited number of trials. Rezq's study^14^ showed that the incidence of MI in the Sacubitril/Valsartan group was lower than that in the ACEI group (1% vs. 2%). On the contrary, Wang's study^15^ showed the incidence of MI the Sacubitril/Valsartan group was higher than that in the ACEI group (5.9% vs. 4.3%).

### 3.5 HF hospitalization outcome in patients after AMI

Three trials^14^, ^16^, ^17^ involving 6017 patients reported HF hospitalization outcome. The significant heterogeneity was noted between the included studies (*I* ^2^ = 69.0%, *P* = .040). However, the meta regression, subgroup analysis or sensitivity analysis could not be performed to analyze the causes of heterogeneity due to the limited number of trials. Therefore, the random‐effects I‐V model was used. The meta‐analysis showed no differences in the incidence of HF hospitalization between the Sacubitril/Valsartan group and the ACEI group (RR: 0.62, 95% CI: 0.37–1.03, *P* = .065; Figure 2(B)).

### 3.6 MACE outcome in patients after AMI

Three studies^14^, ^15^, ^16^ involving 493 patients reported the MACE outcome. The significant heterogeneity was not noted between the included studies (*I* ^2^ = 0.0%, *P* = .414). Therefore, the fixed‐effects M‐H model was used. The meta‐analysis showed that the incidence of MACE in the Sacubitril/Valsartan group was lower than the ACEI group (RR: 0.61, 95% CI: 0.46–0.82, *P* = .001; Figure 2(C)).

### 3.7 LVEF outcome in patients after AMI

Three studies^14^, ^15^, ^16^ involving 483 patients reported the LVEF outcome. The significant heterogeneity was not noted between the included studies (*I* ^2^ = 0.0%, *P* = .399). Therefore, the fixed‐effects M‐H model was used. The meta‐analysis showed that the LVEF in the Sacubitril/Valsartan group was higher than the ACEI group (SMD: 0.37, 95% CI: 0.19–0.55, *P* = .000; Figure 2(D)).

### 3.8 Adverse side effects

Three trials^14^, ^15^, ^17^ reported adverse side effects. Rezq's study^14^ showed that no safety adverse events (such as symptomatic hypotension, worsening renal function, or angioedema) were observed between two groups. Thus, a meta‐analysis could not be performed due to the limited number of trials. Wang's study^15^ and Kunbhani's study^17^ suggested that the incidences of adverse side effects in term of cough and hyperkalemia were lower in the Sacubitril/Valsartan group than the ACEI group, while the incidence of hypotension in the Sacubitril/Valsartan group was higher than the ACEI group.

## 4 DISCUSSION

Since the advent of Sacubitril/Valsartan, its benefits for the HFrEF patients and the HF with preserved ejection fraction patients have been confirmed in PARADIGM‐HF study and PARALLAX study, respectively.^18^, ^19^ The activation of RAAS is the main determinant of the pathophysiology of AMI and HF. Considering that Sacubitril/Valsartan could inhibit the activation of RAAS, many researchers hypothesized that Sacubitril/Valsartan have benefits in patients after AMI.^20^, ^21^ For these patients, there were still confusions about the benefits and risks of Sacubitril/Valsartan and ACEI. After a comprehensive search and strict screening, a total of four studies involving 6154 patients were included. The quantity of included studies was limited, the reason might be that the relevant trials are ongoing or the results have not yet been published. PARADISE‐MI, a multinational, double‐blind, active‐controlled trial, randomized patients within 0.5–7 days of presentation with index AMI to Sacubitril/Valsartan or ramipril. The design and baseline characteristics of the PARADISE‐MI trial have been published.^21^ The primary results of the PARADISE‐MI trial were presented at the American College of Cardiology's 70th Annual Scientific Session,^17^ but they have not been formally published in sources such as books or journals. After discussion, the PARADISE‐MI trial has been considered eligible to be included in this study. The results of meta‐analysis showed that the LVEF in the Sacubitril/Valsartan group was higher than ACEI group, the incidence of MACE in the Sacubitril/Valsartan group was lower than the ACEI group, while the incidences of cardiac death and the HF hospitalization showed no difference.

Owing to limited quantity of trials, the Sacubitril/Valsartan group showed no obvious advantage than the ACEI group in this meta‐analysis when comparing the incidences of MI and the adverse side effects. According to the Cochrane Handbook,^22^ the funnel plot should generally not be considered when the included studies were less than 10, therefor we do not use funnel plot to evaluate the publication bias.

All trials had risks of bias in at least one of several key criteria. Two trials^14^, ^17^ reported adequate sequence generation and one trial^15^ reported adequate concealment of allocation. Two trials^15^, ^16^ had the risk of bias due to absence of blinding of participants and personnel and binding of outcome assessment. The GRADE system entails an assessment of the quality of a body of evidence which involves consideration of within‐study risk of bias, directness of evidence, heterogeneity, precision of effect estimates and risk of publication bias for each individual outcome. Although all trials included did not report adequate concealment of allocation, we think that potential limitations were unlikely to lower confidence in the estimate of effect. Since most trials included did not report adequate sequence generation, we think that potential limitations were probably to lower confidence in the estimate of effect. According to that, we downgraded randomized trial evidence of the cardiac death, the HF hospitalization, the MACE and the LVEF outcomes to moderate quality evidence.

### 4.1 Limitations

The present analysis has several limitations. Firstly, four RCTs were included in this study, resulting in a small number of included patients. Secondly, most studies included have not reported the sequence generation and allocation concealment which could result in selection bias. Finally, language restriction could have introduced publication bias.

## 5 CONCLUSION

This meta‐analysis indicated that early initiation of Sacubitril/Valsartan in patients after AMI was superior to ACEI in reducing the risks of MACE and increasing LVEF, while it had no obvious advantage in reducing the risks of cardiac death, HF hospitalization, MI and adverse side effects.

## CONFLICT OF INTEREST

All authors declare that they have no competing interests.

## AUTHOR CONTRIBUTIONS

Jing Zhao: data curation, formal analysis, writing‐original draft. Yuanyuan Zeng: data curation. Xiaoxu Shen: writing‐review and editing.

## Data Availability

The data supporting this meta‐analysis are from previously reported studies and datasets, which have been cited.
